# Report of the Experiments on Animal Magnetism, &c. Translated, with an Historical and Explanatory Introduction, and an Appendix

**Published:** 1834-04-01

**Authors:** 


*Report of the Experiments on Animal Magnetism, made by a Committee of the Medical Section of the French Royal Academy of Sciences; read at the Meetings of the* 21*st and* 28*th of June*, 1831. *Translated, and now for the first time published, with an Historical and Explanatory Introduction, and an Appendix.* By J. C. Colquhoun, Esq.—Edinburgh, 1833. 8vo. pp. 252.

The author of this work (a Scottish barrister, we believe,) is an enthusiastic magnetiser,—that is to say, on paper; for we do not find that he has magnetised, or been magnetised, himself; but he has collected and narrates everything relating to this curious art in a clear style and gentlemanly tone of feeling. He thinks that traces of a knowledge of the art are to be found in the customs and rites of the most remote ages, and that something very like the modern practice is mentioned by one of the oldest of the Greek poets:

"In the following verses of Solon, we have the earliest and perhaps the directest testimonies to the practice of manipulation, as a sanative process, to be found in antiquity. It is surprising that they should have hitherto escaped the notice of all the writers upon animal magnetism, many of whom have exercised great diligence in collecting the allusions to this process which occur among the ancients.

*Πολλάκι δ' ἐξ ὀλίγης ὀδύνης μέγα γίγνεται ἄλγος,*
*Κοὐκ ἂν τίς λύσαιτ' ἤπια φάρμακα δούς.*
*Τὸν δὲ κακαῖς νούσοισι κυκώμενον ἀργαλέαις τε*
*Α῾ψάμενος χειροῖν, ἄιψα τίθησ' ὑγιῆ.*
*Solon, apud Stobæum.** (P. 35.)

Passing over the whole of the introduction, which is replete with interesting matter, we come to the body of the work itself. It appears that some years since M. Foissac addressed a letter to the Academy of Medicine, requesting that an inquiry might be made into the phenomena of animal magnetism. A committee was appointed in pursuance of his request, February 28, 1826: it consisted of MM. Bourdois, Double, Itard, Gueneau de Hussy, Guersent, Fouquier, Laennec, Leroux, Magendie, Marc, and Thillaye. M. Laennec being obliged to leave Paris, M. Huson was substituted in his stead; MM. Magendie and Double did not attend the experiments. The committee appear to have carried on their investigations for about five years, with every variety of success and failure. Some of the successful cases admit of an easy explanation: the parties may have been tutored, or may have feigned an insensibility to noises, &c.; but there are others, again, which do not admit any solution of this kind. For instance, a man named Paul exhibited what the magnetists call *clairvoyance* in the presence of the committee:

"While his eyelids were kept closed by M. Segalas, there was presented to him a volume which the reporter had brought along with him. He read upon the title-page, *Histoire de France.* He could not read the two intermediate lines, and upon the fifth he read only the name, Anquetil, which is preceded by the prepo-

* "Stanley, in his History of Philosophy, (1666,) has given us a very competent translation of these verses:

"The smallest hurts sometimes increase and rage
More than all art of physic can assuage;
Sometimes the fury of the worst disease
The hand, by gentle stroking, will appease."

sition *par.* The book was opened at the 89th page, and he read in the first line—*le nombre de ses*—he passed over the word *troupes,* and continued: *Au moment ou on le croyait occupé des plaisirs du carnaval.* He also read the running title *Louis*, but could not read the Roman cipher which follows it. A piece of paper was presented to him, upon which were written the words, *Agglutination* and *Magnetisme Animal.* He spelt the first, and pronounced the two others. Finally, the *procès-verbal* of this sitting was presented to him, and he read very distinctly the date, and some words which were more legibly written than the others. In all these experiments the fingers were applied to the whole of the commissure of both eyes, by pressing down the upper upon the under eyelid, and we remarked that the ball of the eye was in a constant rotatory motion, and seemed directed towards the object presented to his vision." (P. 167.)

We hasten to conclude this short notice, lest we should seem to many of our readers to occupy too much space in a practical Journal with the details of what they may consider the "fabric of a vision;" but we can assure them, that if they have confined their reading to the one-sided accounts given by adverse critics, they will be startled by the evidence accumulated in Mr. Colquhoun's book. In short, we agree entirely with Dr. Prichard, who says, "On the whole, when we consider the degree of suffering occasioned by disorders of the class over which magnetism exerts an influence through the medium of the imagination, and the little efficacy which ordinary remedies possess of alleviating or counteracting them, it is much to be wished that this art, notwithstanding the problematical nature of the theories connected with it, were better known to us in actual practice, and that some of the foreign operators would introduce it more extensively into this country." (*Cyclopædia of Pract. Med.*, art. *Somnambulism.*)

